# Multi-phase coronary magnetic resonance angiography using a 3D cones trajectory

**DOI:** 10.1186/1532-429X-13-S1-P238

**Published:** 2011-02-02

**Authors:** Holden H Wu, Bob S Hu, Dwight G Nishimura, Michael V McConnell

**Affiliations:** 1 Stanford University, Stanford, CA, USA; 2 Palo Alto Medical Foundation, Palo Alto, CA, USA

## Introduction

3D whole-heart free-breathing coronary MRA simplifies prescription effort, requires less patient cooperation, and supports retrospective reformats at arbitrary planes. However, this technique can require long scan times and must account for respiratory and cardiac motion.

## Purpose

**Figure 1:**
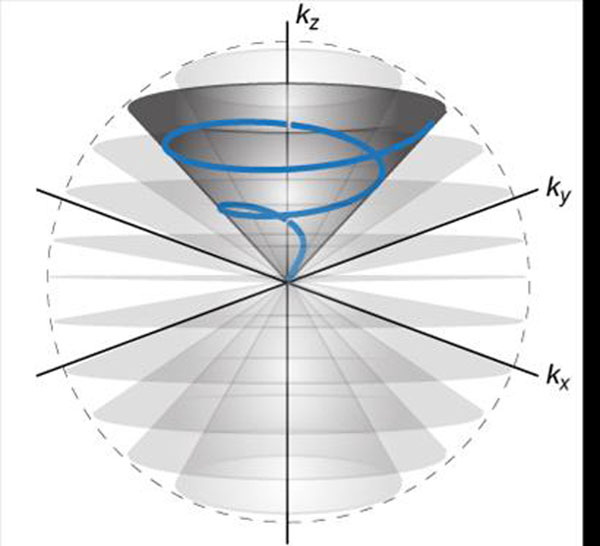
The 3D cones trajectory covers a spherical volume in *k*-space with a series of nested conical surfaces. Each surface is sampled by spiraling readouts that start from the center of *k*-space.

To reduce the scan time and improve the motion robustness for 3D whole-heart free-breathing coronary MRA by using the 3D cones readout trajectory (Fig. 1) [1,2] combined with 2D spiral navigators and resolving multiple cardiac phases.

## Methods

**Figure 2:**
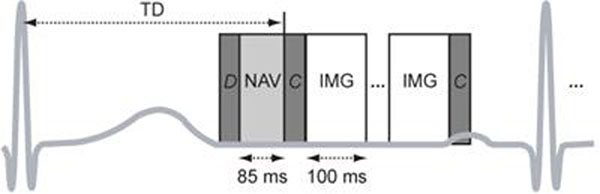
Triggered pulse sequence. TD: delay to start of diastole, D: dummy cycles for navigator imaging, NAV: 2D spiral navigator, C: catalyzation cycles for 3D cones imaging, IMG: 3D cones acquisition for one cardiac phase.

Axial slabs covering the whole heart were imaged on a GE Signa 1.5 T Excite system using a surface coil. Following detection of the cardiac trigger and delay TD (Fig. 2), a 2D sagittal spiral navigator image (3-mm resolution) containing the left ventricle was acquired for respiratory motion tracking. Ten catalyzation cycles were then played out to establish the steady state for 3D cones imaging, which was implemented in an alternating-TR SSFP sequence (TR_total_ = 5.5 ms) [3] to achieve fat suppression and blood-myocardium contrast. The 3D cones trajectory in this experiment supported a FOV of 24x24x16 cm^3^ and resolution of 1.2x1.2x1.25 mm^3^ using 8942 readouts (3-fold acceleration vs. 3D Cartesian), where 18 readouts were acquired per segment (100 ms) and repeated for 2 cardiac phases each heartbeat. Scan time for a single pass was 497 heartbeats and 2 passes were acquired to support retrospective navigator gating (+/-1.5 mm S/I window). Accepted readouts were corrected for 2D displacement (S/I and A/P) and used for 3D gridding reconstruction. The acquisition scheduling scheme supported sliding window reconstruction of multiple intermediate cardiac phases.

## Results

**Figure 3:**
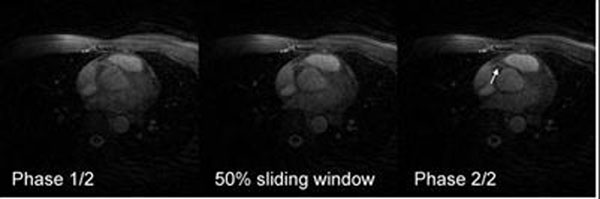
Axial slice containing the RVA after navigator gating and correction for 2D displacement. Shown are the two resolved cardiac phases and an intermediate phase obtained from sliding window reconstruction. In this case, the RCA (arrow) sharpens as the cardiac cycle progresses from phase 1 to 2.

Fig. 3 shows an axial slice containing the right coronary artery (RCA) obtained from one healthy volunteer, reconstructed at the two fully-resolved cardiac phases and one intermediate phase. The RCA sharpens significantly as the cardiac cycle progresses from phase 1 to 2.

## Conclusions

The 3D cones whole-heart free-breathing coronary MRA technique reduces scan time and improves robustness to motion. 2D navigator images directly measure respiratory motion of the heart and provide robust motion correction even without navigator gating. Multiple resolved cardiac phases provide robustness to the initial choice of TD and subsequent heart-rate variations. Additional cardiac phases can be acquired to optimize the visualization of the left and right coronary trees, which may have different quiescent periods.
